# Targeted elimination of *Vancomycin* resistance gene *vanA* by CRISPR-Cas9 system

**DOI:** 10.1186/s12866-023-03136-w

**Published:** 2023-12-04

**Authors:** Shuan Tao, Chunwei Hu, Yewei Fang, He Zhang, Yao Xu, Lin Zheng, Luyan Chen, Wei Liang

**Affiliations:** 1 Department of Clinical Laboratory, The First Affiliated Hospital of Ningbo University, No 59. Liuting Road, Haishu District, Ningbo, 315010 China; 2 School of Medicine, Jiangsu University, Zhenjiang, China; 3 The Biobank of The First Affiliated Hospital of Ningbo University, Ningbo, China; 4 Bengbu Medical College, Bengbu, China; 5 School of Medicine, Ningbo University, Ningbo, China; 6 Department of Blood Transfusion, The First Affiliated Hospital of Ningbo University, Ningbo, China

**Keywords:** CRISPR-Cas9, *Enterococcus*, Antimicrobial resistance, *vanA*

## Abstract

**Objective:**

The purpose of this study is to reduce the spread of the v*anA* gene by curing the *vanA*-harboring plasmid of vancomycin-resistant using the CRISPR-Cas9 system.

**Methods:**

Two specific spacer sequence (sgRNAs) specific was designed to target the *vanA* gene and cloned into plasmid CRISPR-Cas9. The role of the CRISPR-Cas system in the plasmid elimination of drug-resistance genes was verified by chemically transformation and conjugation delivery methods. Moreover, the elimination efficiency in strains was evaluated by plate counting, PCR, and quantitative real-time PCR (qPCR). Susceptibility testing was performed by broth microdilution assay and by Etest strips (bioMérieux, France) to detect changes in bacterial drug resistance phenotype after drug resistance plasmid clearance.

**Results:**

In the study, we constructed a specific prokaryotic CRISPR-Cas9 system plasmid targeting cleavage of the *vanA* gene. PCR and qPCR results indicated that recombinant pCas9-sgRNA plasmid can efficiently clear *vanA*-harboring plasmids. There was no significant correlation between sgRNA lengths and curing efficiency. In addition, the drug susceptibility test results showed that the bacterial resistance to vancomycin was significantly reduced after the *vanA*-containing drug-resistant plasmid was specifically cleaved by the CRISPR-Cas system. The CRISPR-Cas9 system can block the horizontal transfer of the conjugated plasmid pUC19-*vanA*.

**Conclusion:**

In conclusion, our study demonstrated that CRISPR-Cas9 achieved plasmid clearance and reduced antimicrobial resistance. The CRISPR-Cas9 system could block the horizontal transfer of plasmid carrying *vanA*. This strategy provided a great potential to counteract the ever-worsening spread of the *vanA* gene among bacterial pathogens and laid the foundation for subsequent research using the CRISPR-Cas9 system as adjuvant antibiotic therapy.

## Introduction

The overuse and misuse of antibiotics have drastically accelerated the development and spread of antibiotic-resistance genes (ARGs) and multidrug-resistant pathogens [1, 2]. The acquisition and spread of antimicrobial resistance-associated genes have been largely attributed to the role of mobile genetic elements (MGE) such as plasmids, integrons, insertion sequence (ISs), transposons (Tns), and integrated junction elements (ICE) [3]. The horizontal gene transfer (HGT) of ARGs mediated by MGEs aggravates the infection and increases difficulties in clinical treatment [4]. Therefore, non-antibiotic prevention and control bacterial resistance technology has become a research hotspot in the biomedical field in various countries.

*Enterococcus* is an opportunistic pathogen that is one of the main pathogens of nosocomial infections with high morbidity and fatality rates [5]. Enterococci are often naturally resistant to a variety of antibiotics. In addition, enterococci can acquire drug resistance by horizontal gene transfer [6, 7]. In recent years, the isolation of resistant enterococci has increased, which hampers access to efficient treatments [8]. It has been found that the wide spread of mobile gene elements such as transposon is a key factor in the rapid spread of vancomycin-resistant enterococci (VRE) [9, 10]. The intensity of treatment, multiple drug resistance, and nosocomial transmission of VRE strains pose challenges to clinical treatment [11]. Therefore, it is necessary to discover and develop novel antimicrobial strategies to combat the wide dissemination of vancomycin resistance genes *vanA* and limit the spread of plasmid-borne resistance.

Clustered regularly interspaced short palindromic repeat (CRISPR)-associated (CRISPR-Cas) systems are adaptive defense systems that protect bacteria and archaea from invading genetic elements. [12] CRISPR-Cas systems are considered as barriers to horizontal gene transfer (HGT) [13]. CRISPR-Cas is reported to re-sensitize bacterial resistance to antibiotics by specifically targeting and eliminating the plasmids carrying antibiotic resistance genes [14, 15]. Type II CRISPR-Cas9-mediated gene editing has potential applications in the prevention and control of the spread of bacterial drug resistance, which can be used to combat contagious infections and develop novel antimicrobial drugs [16].

In the present study, we constructed a CRISPR-Cas9 system targeting *vanA*-carrying plasmids that mediate bacterial resistance to *vancomycin* antibiotics, in an attempt to re-sensitize drug-resistant bacteria by eliminating drug-resistant plasmids. The results demonstrated that the prokaryotic CRISPR-Cas9 system can eliminate the *vanA* plasmid in drug-resistant model bacteria and reduce the resistance to vancomycin by removing the resistant plasmids, which offered a novel strategy to combat the dissemination of antibiotic resistance genes among bacterial pathogens at the molecular level and maximize the advantages of CRISPR technology in the field of anti-bacterial resistance.

## Materials and methods

### Bacterial strains, plasmids, and growth conditions

Bacterial strains and plasmids used or constructed in this study are listed in Table 1. *E. coli* DH5 and *E. coli* BL21 were used to make chemically competent cells and strains for plasmid transformation experiments and propagation. The *vanA* resistance genes were obtained from VRE isolated and stocked in this laboratory. The pCas9 plasmid carrying the Cas9 endonuclease and sgRNA-binding site and tracRNA (Addgene, plasmid number 42,876) was obtained from Hunan Fenghui Biotechnology Co., Ltd [17]. *E. coli* has grown in Luria-Bertani (LB, 5 g yeast extract, 5 g NaCl, and 10 g tryptone per liter) broth or on LB agar (LB supplemented with 15 g agar per liter) plates at 37 °C. For plasmid maintenance, Kanamycin(25 mg/mL) and chloramphenicol (50 mg/L) were added when necessary.

**Table 1:** Bacterial strains and plasmids used in this study

Bacterial strains and plasmids		Reference or source
Bacterial strains *E. coli* DH5α	F-,φ80dlacZΔM15, Δ(lacZYA-argF)U169, deoR, recA1, endA1, hsdR17(rk-, mk+), phoA, supE44, λ-, thi-1, gyrA96, relA1	This study
*E.coli* BL21	F-, ompT, hsdSB (rB-mB-) gal,dcm araB::T7RNAP-tetA	This study
VR-*E. faecium*24	*Enterococcus* clinical strains containing vanA	This study
Plasmids pCas9 (Addgene,42,876)	Cmr, tracRNA, gRNA, and cas9 expression plasmid;	Laboratory stock [15]
pCas9-sgRNA	Cmr, pCas9 cloned with sgRNA targeting vanA;	This study
pUC19 (Invitrogen, Carlsbad, CA, USA)	AMPr, expression vector	Laboratory stock
pUC19-vanA	AMPr, recombinant vector derivative with vanA gene	This study

### Plasmid construction

The plasmids were constructed using standard molecular biology techniques [18]. The *vanA* gene was specifically amplified from VRE-*E. faecium*24 with polymerase chain reaction (PCR). DNA segment containing the promoter and *vanA* gene was amplified from *Enterococcus* clinical strains using primers SacI-*vanA*-F/R (SacI-F: 5’-GAGCTCCTACTCTGATTGCACC-3’ and SacI-R: 5’-GAGCTCAAAGTAAATCCTATTTCCA-3’) followed by digestion with SacI and ligated into the corresponding site in pUC19, yielding pUC19-*vanA*. The sgRNAs targeting *vanA* genes were designed by the CHOPCHOP web tool (https://chopchop.cbu.uib.no/). Then their reverse complementary oligonucleotides were synthesized. According to the spacer cloning site requirements of pCas9, ‘AAAC’ overhang at the 5’-end, and a ‘G’ is added at the 3’end of the forward oligos and a 5’AAAAC to the 5’end of the reverse complement oligo. (sgRNA-F: 5’-AAACTGTGAAAAAAGTCAATAGCG-3’ and sgRNA-R:5’-AAAACGCTATTGACTTTTTTCACA-3’). Then the complementary oligonucleotides were annealed and cloned into pCas9 digested with BsaI using T4 DNA ligase (NEB, Ipswich, MA, USA) generating the pCas9-sgRNA recombinant vector. Colony PCR and subsequent sequencing were used to verify the constructed recombinant plasmids. Following the same strategy mentioned above for pCas9-sgRNA(20 bp), we also constructed the pCas9-sgRNA(30 bp).

### Transformation experiments

The competent cells including *E. coli* DH5α containing pET24-vanA were prepared and used for transformation assay following the protocol. The chemical transformation was performed by mixing 100µL of chemically competent cells with 10µL of plasmid pCas9-sgRNA incubated on ice for 30 min, followed by a heat shock for the 90s at 42 °C and finally incubated on ice for 2 min. Then transformed cells were grown in 1 ml LB media and incubated at 37 °C, with vigorous shaking at 200 rpm for 1 h. The putative transformant strains were selected on LB agar containing chloramphenicol (50 mg/L) and cultured at 37 °C overnight. The empty plasmid pCas9 was used as the negative control.

### Evaluation of the CRISPR-Cas Elimination Efficiency

#### PCR detection of *vanA* plasmid clearance efficiency

After transformation, 20 single clones were randomly selected to evaluate the elimination efficiency. The CRISPR plasmid was detected by PCR with primers pCas9-F/R(pCas9-F: CGGCGTTATCACTGTATTGCACGG and pCas9-R: TGTGTACGCGATGGATACCG). At the same time, the colonies were screened for target gene deletions by PCR with primers *vanA*-F/R (*vanA*-F: CGAGCCGTTATACATTGGA and *vanA*-R: CATATTGTCTTGCCGATTCA). The strain carrying the *vanA* gene was used as a positive control.

#### E-test antimicrobial test strips detect bacterial resistance phenotype

Antimicrobial sensitivity assays were performed using Etest strips (bioMérieux, Sweden) according to standard operating procedures on colonies with negative PCR product bands and positive control colonies. MIC of vancomycin was obtained by E-test strips according to the guidelines of the Institute for Clinical and Laboratory Standards (2019).

#### Quantitative PCR (qPCR) detection of *vanA* copy number changes

To further analyze the efficiency of eliminating the *vanA* gene, the genomic DNA of experimental group pCas9-sgRNA(20 and 30 bp) and control groups pCas9 were extracted using TIANamp Bacteria DNA Kit (Tiangen, Beijing, China). The change of pUC19-vanA copy numbers at 2,4,8,16, and 32 h after transformation into the CRISPR-Cas9 system were calculated using the SYBR Green fluorescent dye of fluorescent quantitative PCR (qPCR) with primers specific for *vanA* gene (vanA-F: CAAGTCAGGTGAAGATGGAT and *vanA*-R: CGCAACGATGTATGTCAAC) and using the chromosomal 16 S ribosomal RNA (16 S rRNA) gene(16 S-F: AGAGTTTGATCCTGGCTCAG and 16s-R:CTGCTGCCTCCCGTAGGAGT) as the internal reference. Reactions were conducted in 20µL volume reactions comprising: 2 µL of DNA, 0.4ul of each above corresponding primer, and 10 µL of SYBR Green PCR master mix ((Biomed, Beijing, China). Non-template controls were included in each analysis plate to monitor possible reaction contamination [19]. RT-PCR amplification was performed using three-steps method. Pre-denaturation: 1 cycle (95 °C for 2 min); PCR reaction: 40 cycles (95 °C for 15 s, 60 °C for 30 s) and dissolution curve (1 min at 95 °C, 1 min at 65 °C), and a gradual temperature increase from 65 to 95 °C at 2 °C/min. All reactions were run in triplicate. Primer amplification efficiencies were determined by the Ct slope method; efficiencies for the above primer pairs were comparable (90%) and no amplification was detected in the no template control. Relative gene expression of *vanA* copy number in the experimental group compared to the control group was calculated by the 2^−ΔΔCT^ CT method [20].

### Conjugation assays

Conjugation experiments were performed with *E. coli* C600 + pUC19-*vanA* as the donor and *E. coli* C600 + pCas9-sgRNA or C600 + pCas9 as the recipient strain [21]. Donor and recipient strains were grown in suspension overnight, then diluted 1:100 in fresh LB broth and incubated at 37 until OD 600 to 0.4. Then, the donor/recipient ratio was 1:4 (vol/vol), mixed, and incubated for 24 h. Serial dilutions of the mixture were placed on LB agar plates supplemented with chloramphenicol and kanamycin to select the transconjugates. After 24 h of incubation, the number of colony forming units (CFU) was determined.

### Statistical analysis

Statistical analysis was performed with GraphPad Prism version 9.0 (GraphPad Software Inc., San Diego, CA, USA). Normality was assessed using skewness and kurtosis statistics. P values were calculated using the Student’s t-test and P < 0.05 was considered as statistical significance.

## Results

### Construction of the CRISPR-Cas9 plasmid and recombinant plasmid targeting the *vanA* gene

**Fig. 1:**
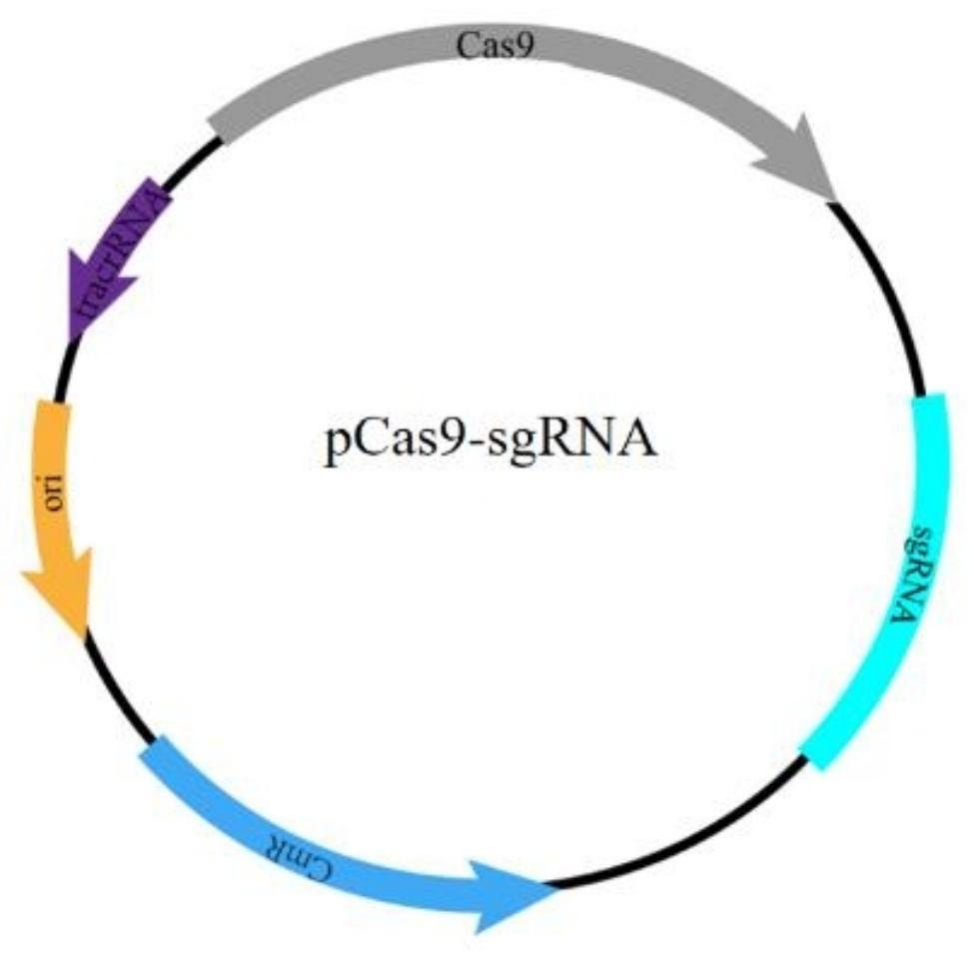
Plasmid map of pCas9-sgRNA targeted to *vanA* gene. The pCas9-sgRNA was constructed by inserting spacer targeting the *vanA* gene for CRISPR-Cas9 activity

According to the NGG PAM sequence (protospacer adjacent motif sequence) principle [22], the sgRNAs were designed for *vanA* in the promoter region or near the ATG sequence in the targeted gene and were right after an NGG sequence. Then the oligonucleotide with 20nt and 30nt was cloned into the pCas9 plasmid and obtained pCas9-sgRNA specifically targeting *vanA* (Fig. 1). In addition, we selected the high-copy plasmid pUC19 as the backbone to construct a recombinant plasmid pUC19- *vanA* containing the *vanA* gene.

### Validation of the effect of CRISPR-Cas9 cutting *vanA* plasmid

**Fig. 2:**
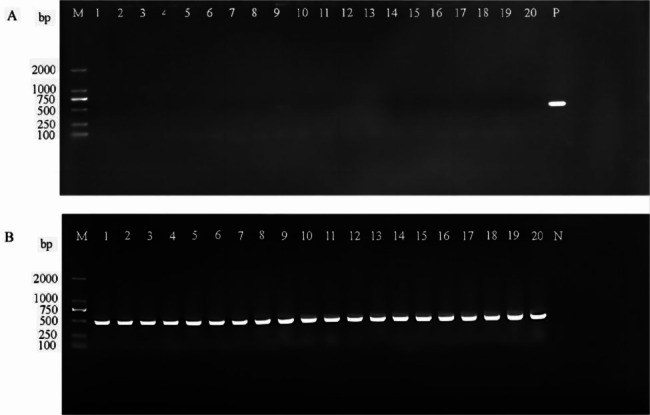
PCR amplification using primers vanA-F/R to identify pCas9-sgRNA elimination of *vanA* gene in *E. coli DH5α* + pUC19-*vanA*. (A) The *E.coli* DH5α + pUC19-*vanA* strain transformed with pCas9-sgRNA (B) and the *E.coli* DH5α + pUC19-*vanA* strain transformed with pCas9. Markers with an M value of 2,000 bp. P represents the *E. coli* DH5α + pUC19-*vanA* strain in which pCas9 is a positive control. N indicates a negative control. Markers with an M value of 2,000 bp. P represents the *E. coli* DH5α + pUC19-*vanA* strain in which pCas9 is a positive control and N denotes negative control

To assess the efficiency of the CRISPR-Cas9 system in mediating plasmid elimination in *E.coli*, the control vector pCas9 and the engineered vector pCas9-sgRNA(20 bp) were transformed into competent *E. coli* BL21 + pUC19-*vanA*. Then 20 single colonies were selected from the plates of the experimental group and the plates of the control group, respectively. The results showed that all 20 colonies in the experimental group were negative for *vanA*, indicating the absence of *vanA* gene by PCR after specific cleavage by Cas9 nuclease. The shear success rate was 100% in the selected 20 transformants. In contrast, the Cas9 nuclease in the control group lacked the guidance of sgRNA and showed no specific cleavage of *vanA* (Fig. 2). Similarly, the colony PCR results indicated that the efficacy of pCas9-sgRNA(30 bp) was not significantly different from that of pCas9-sgRNA(20 bp). The PCR results indicate that the specific CRISPR system could cut and destroy the *vanA* gene.

### Changes in bacterial phenotypes

The results of Etest strips found the level of resistance to vancomycin in the experimental group was significantly reduced by about 4 times after *vanA* was specifically cleaved by the CRISPR-Cas9 system. (MIC > 256 mg/L vs. 64ug/ml).

### Analysis of CRISPR System Editing Efficiency of *vanA*

**Fig. 3:**
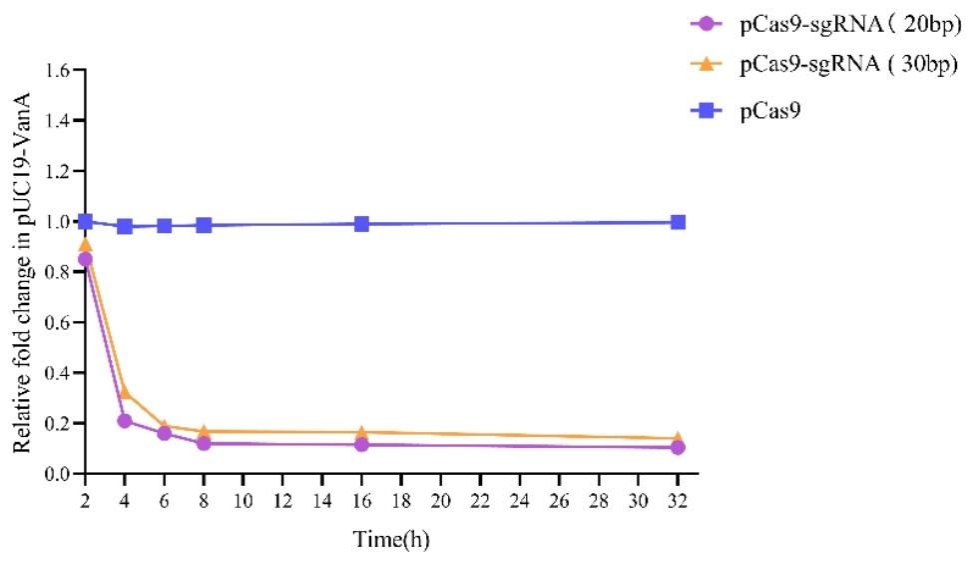
Relative copy number of the plasmid pUC19-*vanA* (20 and 30 bp) at each time point. pCas9-sgRNA (20 and 30 bp) and pCas9 were transformed into competent *E.coli C600* + pUC19-*vanA* as the experimental group and the control group, respectively. The sgRNA (20 and 30 bp) are the lengths of the sgRNA targeting the *vanA* resistance gene

In this study, Quantitative PCR (qPCR) analysis of the efficiency of pCas9-sgRNA in clearing *vanA* was performed to calculate the relative copy number of plasmids at each time point in the pCas9-sgRNA(20 or 30 bp) experimental group and the pCas9 control group after transformation of the CRISPR-Cas9 system (Fig. 3). The quantitative analysis found that there were significant differences in plasmid copy number between the experimental and control groups. The experimental group cleared about 80% of the resistant plasmid at 4 h and the clearance of the resistant plasmid continued until the 32nd hour.

### The CRISPR-Cas9 system can block plasmid conjugation

**Fig. 4:**
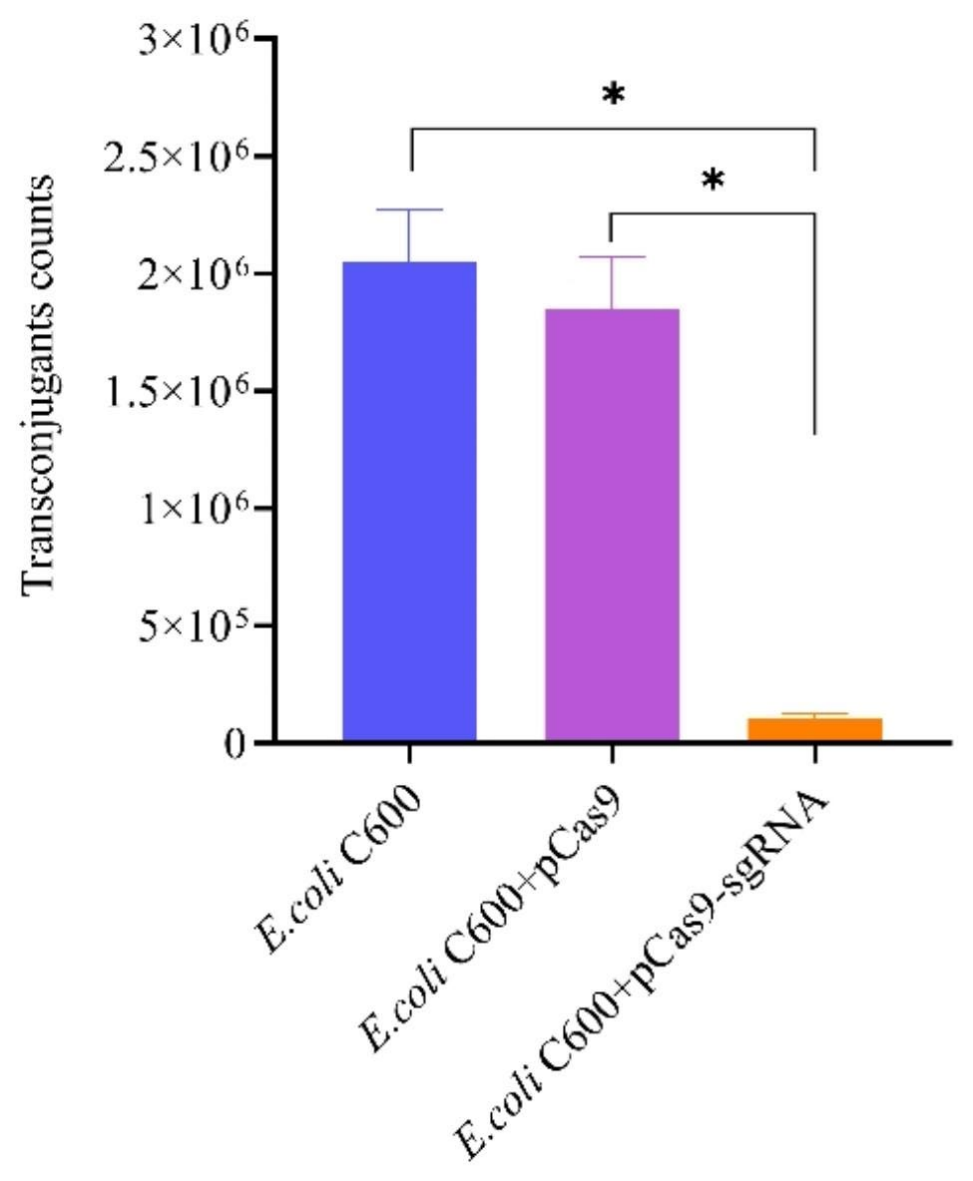
*E. coli C600* containing the pCas9-sgRNA(20 bp) eliminated the *vanA* resistance genes. *E. coliC600 +* pUC19-*vanA* served as the donor, and *E. coli C600* + pCas9-sgRNA(20 bp) or *E. coli C600* + pCas9 served as the recipient strain. All bar graphs represent the mean of at least three biological replicates. Errors bars represent standard errors from three biological replicates. *p < 0.05

*E.coli* C600 containing pCas9-sgRNA(20 bp) and pCas9 were used as recipient cells. *E. coli* C600 carrying the coupled plasmid pUC19-vanA was used as the recipient cell. The number of transconjugates in *E. coli* C600 containing the plasmid pCas9-sgRNA(20 bp) was reduced by 4-fold compared with the control plasmid pCas9 (Fig. 4).

## Discussion

The HGT-mediated acquisition of ARGs is largely responsible for the spread of antimicrobial resistance [4]. The emergence of vancomycin-resistant *enterococcus* brings challenges to the clinical prevention and control of infection caused by clinical pathogens [23]. The restoration of the sensitivity to traditional antibiotics might be more effective than searching for new antimicrobials. Therefore, new strategies for the prevention and control of the dissemination of VRE should be developed.

In this study, we investigated the potential of the CRISPR-Cas9 system to counteract plasmid-borne *vanA* gene high-copy numbers of plasmids (pUC19-vanA). The results showed that the CRISPR-Cas9 system could effectively eliminate the *vanA* gene. Although the *vanA* plasmid has the potential to restore its copy, this experiment confirmed that no rebound in the copy amount of the drug-resistant plasmid was observed during the process of CRISPR clearing *vanA*. Moreover, the CRISPR-Cas9 system could effectively remove the vancomycin resistance plasmid by targeting the *vanA* gene, and the rate of vancomycin resistance decreased after the removal of the plasmid. Compared with other similar studies [24], the CRISPR-Cas9 system in this study achieved plasmid clearance and reduced resistance to vancomycin rather than simultaneously re-sensitizing to vancomycin, possibly due to the model bacteria – *E.coli* natural resistance to glycopeptide. Some studies have pointed out that there are differences in the efficiency of CRISPR-Cas9 system editing in wild plasmids and model plasmids [24, 25]. It may be because we speculate that the high-copy plasmid is an engineered plasmid with a clear plasmid background, while the wild-type plasmid has been clinically isolated and has a more complex background. Bacterial conjugation is critical to the spread of antimicrobial resistance, and conjugation plasmids were explored to eliminate antibiotic resistance plasmids in bacteria [26]. Our study indicated that the *vanA*-harbouring plasmids in the recipient can be eliminated via conjugative delivery of the CRISPR-Cas9 system. Furthermore, the conjugative delivery of CRISPR-Cas9 antimicrobials may be suitable for the precise targeting of established multidrug-resistant bacteria. Our current research mainly employs CRISPR-Cas to counteract antibiotic resistance mediated by the *vanA* gene harbored in the high-copy plasmid pUC19. The follow-up research will focus on application in wild plasmid and clinical isolates.

Studies related to CRISPR gene editing have reported that good sgRNA design can optimize the shearing efficiency of the CRISPR system [27, 28]. Especially in eukaryotic systems, different target sites can lead to differences in CRISPR shear efficiency, as well as different degrees of off-target effects [29, 30]. It has been indicated that the sgRNA sequence of different lengths may affect the curing efficiency of the plasmid. Zhang et al. [31] showed that knockout sgRNA with the potency of 17nt or 20nt varied in different host stem cells and this difference could be explained by the differential levels of Cas9 expression in different cell types. However, it has also been shown that the knockdown efficiency of 17nt sgRNA in human cells is similar to that of 20nt sgRNA, but its off-target mutagenic effect is greatly reduced. This may indicate that the effect of sgRNA sequence length varies from host to host [32].

However, the existence of extensive multidrug resistance (MDR) may be unsatisfactory to eliminate using a single non-essential target. Therefore, the efficiency of the CRISPR-Cas system can be improved by designing a CRISPR-Cas system to target the essential genes on the resistance plasmid or using a CRISPR array to establish multiple cleavage sites simultaneously. Studies have shown that two sgRNAs on a single structure can be used to target and remove *bla*_NDM−1_ and *bla*_CTX−M−15_ genes [33], which is important for reducing the spread of MDR. Rodriguez et al [34] used the CRISPR-Cas9 system to target the *tet(M)* and *erm(B)* genes conferring resistance to tetracycline and erythromycin, respectively and successfully reduced the drug resistance of *Enterobacter faecalis in vitro and in vivo*. Hao et al [35]. developed the plasmid curing system pCasCure based on the CRISPR-Cas9 system to precisely cut and define carbapenemase genes such as bla_*NDM*_, bla_*KPC*_, bla_*OXA−48*_ carbapenem-resistant Enterobacteriaceae (CRE) and targeted Prevalence of repA, repB, and parApKpQIL plasmids to remove plasmids carrying carbapenemase resistance genes and re-sensitive CRE carbapenem antibiotics. The MIC value was reduced by more than 8 times. Scientists are attempting to develop a CRISPR-Cas9 system to restore antibiotic susceptibility of extended-spectrum-lactamase (ESBL)-producing *E. coli* by identifying a conserved target sequence among > 1000 ESBL mutants [36]. Therefore, combining experimental and analytical methods, designing the best sgRNA can improve the activity and specificity of sgRNA.

In addition, the efficacy of CRISPR-Cas9 mainly depends on the delivery efficiency of the system [37]. And since the transformation is a non-spontaneous mode, this form may be limited in clinical application. Therefore, other delivery methods such as plasmid conjugation, and phage methods have been used *in vitro or in vivo* models [38, 39]. Previous studies have found that it can be overcome by using nanomaterials as non-viral vectors to deliver the CRISPR-Cas9 system [40]. A variety of innovative polymers, lipids, and gold nanoparticles have been developed [41]. However, the integration of materials with CRISPR systems is still in its early stages. The focus of the subsequent research is on how to efficiently present the CRISPR-Cas9 system to the bacterial cells so that the system can autonomously spread, and eliminate the drug resistance genes carried in the environmental microbiota by spreading the CRISPR-Cas9 system to the flora, and combat the spread of bacterial drug resistance, to curb the spread of bacterial drug resistance in various regions. The successful application of the CRISPR-Cas system in the treatment of bacterial infections and the control of the spread of drug-resistant bacteria requires further research.

## Conclusion

In conclusion, we demonstrated the prokaryotic CRISPR-Cas9 system eliminates *vanA* genes and plasmids to interrupt the HGT of ARGs. The strategy provides a reference for the active prevention and control of the dissemination of *vanA* resistance.

## Data Availability

The datasets used and analyzed during the current study are available from the corresponding author on reasonable request

## References

[1] Bjerketorp J, Levenfors JJ, Nord C, Guss B, Öberg B, Broberg A (2021). Selective isolation of Multidrug-Resistant Pedobacter spp., producers of novel antibacterial peptides. Front Microbiol.

[2] Qiao M, Ying GG, Singer AC, Zhu YG (2018). Review of antibiotic resistance in China and its environment. Environ Int.

[3] Algarni S, Ricke SC, Foley SL, Han J (2022). The dynamics of the Antimicrobial Resistance Mobilome of Salmonella enterica and related enteric Bacteria. Front Microbiol.

[4] von Wintersdorff CJ, Penders J, van Niekerk JM, Mills ND, Majumder S, van Alphen LB (2016). Dissemination of Antimicrobial Resistance in Microbial ecosystems through horizontal gene transfer. Front Microbiol.

[5] Timmler SB, Kellogg SL, Atkinson SN, Little JL, Djorić D, Kristich CJ (2022). CroR regulates expression of pbp4(5) to promote Cephalosporin Resistance in Enterococcus faecalis. mBio.

[6] Hollenbeck BL, Rice LB (2012). Intrinsic and acquired resistance mechanisms in enterococcus. Virulence.

[7] Arias CA, Murray BE (2012). The rise of the Enterococcus: beyond Vancomycin resistance. Nat Rev Microbiol.

[8] Calfee DP (2012). Methicillin-resistant Staphylococcus aureus and Vancomycin-resistant enterococci, and other Gram-positives in healthcare. Curr Opin Infect Dis.

[9] Courvalin P (2006). Vancomycin resistance in gram-positive cocci. Clin Infect Diseases: Official Publication Infect Dis Soc Am.

[10] Hu Y, Yang X, Li J, Lv N, Liu F, Wu J (2016). The bacterial mobile resistome transfer network connecting the animal and human microbiomes. Appl Environ Microbiol.

[11] Phukan C, Lahkar M, Ranotkar S, Saikia KK (2016). Emergence of vanA gene among Vancomycin-resistant enterococci in a tertiary care hospital of North - East India. Indian J Med Res.

[12] Høyland-Kroghsbo NM, Muñoz KA, Bassler BL. Temperature, by Controlling Growth Rate, regulates CRISPR-Cas activity in Pseudomonas aeruginosa. mBio. 2018;9(6). 10.1128/mBio.02184-18 PMC6234860 30425154

[13] Gabel C, Li Z, Zhang H, Chang L (2021). Structural basis for inhibition of the type I-F CRISPR-Cas surveillance complex by AcrIF4, AcrIF7 and AcrIF14. Nucleic Acids Res.

[14] Gholizadeh P, Köse Ş, Dao S, Ganbarov K, Tanomand A, Dal T (2020). How CRISPR-Cas System could be used to Combat Antimicrobial Resistance. Infect drug Resist.

[15] Shabbir MAB, Shabbir MZ, Wu Q, Mahmood S, Sajid A, Maan MK (2019). CRISPR-cas system: biological function in microbes and its use to treat antimicrobial resistant pathogens. Ann Clin Microbiol Antimicrob.

[16] Pursey E, Sünderhauf D, Gaze WH, Westra ER, van Houte S (2018). CRISPR-Cas antimicrobials: challenges and future prospects. PLoS Pathog.

[17] Su T, Liu F, Gu P, Jin H, Chang Y, Wang Q (2016). A CRISPR-Cas9 assisted non-homologous end-joining strategy for one-step Engineering of Bacterial Genome. Sci Rep.

[18] Sambrook J, Russell DW. Molecular Cloning: A Laboratory Manual, Volume 1. 2001.

[19] Woroszylo M, Ciecholewska-Jusko D, Junka A, Drozd R, Wardach M, Migdal P et al. Rotating magnetic field increases beta-Lactam Antibiotic susceptibility of Methicillin-resistant Staphylococcus aureus strains. Int J Mol Sci. 2021;22(22). 10.3390/ijms222212397 PMC8618647 34830278

[20] Livak KJ, Schmittgen TD (2001). Analysis of relative gene expression data using real-time quantitative PCR and the 2(-Delta Delta C(T)) method. Methods.

[21] Chen L, Chen ZL, Liu JH, Zeng ZL, Ma JY, Jiang HX (2007). Emergence of RmtB methylase-producing Escherichia coli and Enterobacter cloacae isolates from pigs in China. J Antimicrob Chemother.

[22] Ran FA, Hsu PD, Wright J, Agarwala V, Scott DA, Zhang F (2013). Genome engineering using the CRISPR-Cas9 system. Nat Protoc.

[23] Ahmed MO, Baptiste KE, Vancomycin-Resistant Enterococci. A review of Antimicrobial Resistance mechanisms and perspectives of Human and Animal Health. Microbial drug resistance (Larchmont, NY). 2018;24(5):590–606. 10.1089/mdr.2017.0147 29058560

[24] Wan P, Cui S, Ma Z, Chen L, Li X, Zhao R (2020). Reversal of mcr-1-Mediated colistin resistance in Escherichia coli by CRISPR-Cas9 System. Infect drug Resist.

[25] He YZ, Kuang X, Long TF, Li G, Ren H, He B (2021). Re-engineering a mobile-CRISPR/Cas9 system for antimicrobial resistance gene curing and immunization in Escherichia coli. J Antimicrob Chemother.

[26] Álvarez-Narváez S, Giguère S, Berghaus LJ, Dailey C, Vázquez-Boland JA. Horizontal spread of Rhodococcus equi Macrolide Resistance plasmid pRErm46 across Environmental Actinobacteria. Appl Environ Microbiol. 2020;86(9). 10.1128/AEM.00108-20 PMC7170479 32169935

[27] Bai M, Yuan J, Kuang H, Gong P, Li S, Zhang Z (2020). Generation of a multiplex mutagenesis population via pooled CRISPR-Cas9 in soya bean. Plant Biotechnol J.

[28] Xiong Y, Xie X, Wang Y, Ma W, Liang P, Songyang Z (2017). pgRNAFinder: a web-based tool to design distance Independent paired-gRNA. Bioinf (Oxford England).

[29] Liang X, Potter J, Kumar S, Ravinder N, Chesnut JD, Enhanced CRISPR (2017). /Cas9-mediated precise genome editing by improved design and delivery of gRNA, Cas9 nuclease, and donor DNA. J Biotechnol.

[30] Wilson LOW, O’Brien AR, Bauer DC (2018). The current state and future of CRISPR-Cas9 gRNA Design Tools. Front Pharmacol.

[31] Zhang JP, Li XL, Neises A, Chen W, Hu LP, Ji GZ (2016). Different effects of sgRNA length on CRISPR-mediated gene knockout efficiency. Sci Rep.

[32] Fu Y, Sander JD, Reyon D, Cascio VM, Joung JK (2014). Improving CRISPR-Cas nuclease specificity using truncated guide RNAs. Nat Biotechnol.

[33] Yosef I, Manor M, Kiro R, Qimron U (2015). Temperate and lytic bacteriophages programmed to sensitize and kill antibiotic-resistant bacteria. Proc Natl Acad Sci USA.

[34] Rodrigues M, McBride SW, Hullahalli K, Palmer KL, Duerkop BA. Conjugative delivery of CRISPR-Cas9 for the selective depletion of antibiotic-resistant Enterococci. Antimicrob Agents Chemother. 2019;63(11). 10.1128/AAC.01454-19 PMC6811441 31527030

[35] Hao M, He Y, Zhang H, Liao XP, Liu YH, Sun J et al. CRISPR-Cas9-Mediated carbapenemase gene and plasmid curing in Carbapenem-Resistant Enterobacteriaceae. Antimicrob Agents Chemother. 2020;64(9). 10.1128/AAC.00843-20 PMC7449206 32631827

[36] Kim JS, Cho DH, Park M, Chung WJ, Shin D, Ko KS (2016). CRISPR/Cas9-Mediated re-sensitization of antibiotic-resistant Escherichia coli Harboring extended-spectrum β-Lactamases. J Microbiol Biotechnol.

[37] Park JY, Moon BY, Park JW, Thornton JA, Park YH, Seo KS (2017). Genetic engineering of a temperate phage-based delivery system for CRISPR/Cas9 antimicrobials against Staphylococcus aureus. Sci Rep.

[38] Bikard D, Euler CW, Jiang W, Nussenzweig PM, Goldberg GW, Duportet X (2014). Exploiting CRISPR-Cas nucleases to produce sequence-specific antimicrobials. Nat Biotechnol.

[39] Xu S, Pham T, Neupane S (2020). Delivery methods for CRISPR/Cas9 gene editing in crustaceans. Mar life Sci Technol.

[40] Xu X, Liu C, Wang Y, Koivisto O, Zhou J, Shu Y (2021). Nanotechnology-based delivery of CRISPR/Cas9 for cancer treatment. Adv Drug Deliv Rev.

[41] Kong FH, Ye QF, Miao XY, Liu X, Huang SQ, Xiong L (2021). Current status of sorafenib nanoparticle delivery systems in the treatment of hepatocellular carcinoma. Theranostics.

